# Increased blood-cerebrospinal fluid transfer of albumin in advanced Parkinson’s disease

**DOI:** 10.1186/1742-2094-9-188

**Published:** 2012-08-08

**Authors:** Valerio Pisani, Alessandro Stefani, Mariangela Pierantozzi, Silvia Natoli, Paolo Stanzione, Diego Franciotta, Antonio Pisani

**Affiliations:** 1 Department of Neuroscience, University of Rome ‘Tor Vergata’, Rome, Italy; 2 Intensive Care Unit, University of Rome ‘Tor Vergata’, Rome, Italy; 3 Laboratory of Neuroimmunology, IRCCS, National Neurological Institute C. Mondino, Pavia, Italy; 4 Laboratory of Neurophysiology, Santa Lucia Foundation, IRCCS, Rome, Italy

**Keywords:** Albumin ratio, Blood–brain barrier, Blood-cerebrospinal fluid barrier, Cerebrospinal fluid, Parkinson’s disease

## Abstract

**Background:**

Alterations in blood–brain barrier permeability have been proposed to represent a relevant factor contributing to Parkinson’s disease progression. However, few studies have addressed this issue in patients at different stages of disease.

**Methods:**

Albumin was measured in cerebrospinal fluid and serum samples obtained from 73 non-demented subjects with idiopathic Parkinson’s disease and 47 age-matched control subjects. The albumin ratio (AR) was calculated to assess blood-cerebrospinal fluid and blood–brain barrier function. The group of patients with Parkinson’s disease included 46 subjects with Hoehn-Yahr staging between 1 and 2 and 27, with a score ranging from 2.5 to 4.

**Results:**

Statistically significant differences in albumin ratio were found between patients with advanced disease, and both early-stage and unaffected groups. Conversely, early-phase patients did not differ from healthy subjects. Additionally, dopaminergic treatment seems to exert a possible effect on AR values**.**

**Conclusions:**

Our study demonstrates that possible dysfunction of the blood-cerebrospinal fluid barrier, blood–brain barrier, or both, characterize Parkinson’s disease progression. The associations between clinical scores, treatments and biochemical findings suggest a progressive impairment of barrier integrity during the course of the disease.

## Background

A growing body of evidence suggests an important role of inflammation in the pathophysiology of neurodegenerative diseases. Moreover, an impairment of brain barrier filtering systems through direct (leukocyte infiltration, toxins) or indirect (cytokines, growth factors) pathophysiological mechanisms has been proposed in Parkinson’s disease (PD) [1,2]. Particularly, a positron emission tomography (PET) study in PD patients revealed a dysfunction of the blood–brain barrier (BBB) transporter system [3], and increased BBB permeability has also been demonstrated in rat models of PD [4,5]. However, a detailed analysis on inflammation-induced changes of barriers that separate blood from cerebrospinal fluid (CSF) and brain parenchyma in PD is still lacking.

Choroid plexuses, namely the blood-CSF barrier (BCSFB), produce two-thirds of the CSF volume, the remaining one-third deriving from the BBB [6]. The analysis of protein content on lumbar CSF specimens mainly allows the assessment of the functional integrity of the BCSFB, but not the isolated BBB [7,8]. Thus, in attempt to gain a better understanding on the role of BCSFB and BBB function on disease course, we analyzed paired CSF and serum samples and calculated albumin ratio (AR) of PD patients at different disease stages, including both early *de novo* subjects and clinically advanced ones.

## Methods

We screened 102 consecutively admitted and unselected subjects with idiopathic PD according to the UK PD Society Brain Bank criteria [9]. Patients underwent a thorough clinical and neurological evaluation comprehensive of severity assessment (Unified PD Rating Scale (UPDRS) part III and Hoehn-Yahr staging (H&Y)), brain magnetic resonance imaging (MRI), and atraumatic lumbar puncture for CSF analysis. CSF and blood samples were also obtained for diagnostic purposes from 47 age-matched control subjects (CTRL), hospitalized because of complaining non-specific pain, motor or sensory symptoms, though not presenting medical conditions known to impair BCSFB and BBB permeability (Table1). In order to avoid any potential bias, we also added a second independent cohort of 11 subjects (IND) complaining a pharmacoresistant headache, without any evidence for subarachnoid hemorrhage or cerebral neoplasm. Exclusion criteria were: Mini Mental State Examination (MMSE) score <26, evidence of large cortico-subcortical lesions or confluent cerebral infarctions on MRI scans, subjects exhibiting low-back pain or radiculopathies, abnormal CSF cell count (>4 cells/μl), and the presence of intrathecal IgG synthesis, whether quantitatively or qualitatively assessed [8], on routine CSF analysis. All subjects underwent lumbar puncture in the morning of the same day of the clinical evaluation. Patients were punctured lying in lateral position with atraumatic needles. CSF was collected in polypropylene tubes using standard sterile techniques. The first sample (2 ml) was utilized for routine analysis, while a second sample (5 ml) was collected to measure albumin-IgG concentration and oligoclonal bands. Blood specimens were also obtained at the same time of lumbar procedure. Immediately after collection, CSF samples were stored immediately on ice, sent to the local laboratory, and processed within 1 h. Albumin and IgG concentrations were determined by immunonephelometry and oligoclonal bands by isoelectric focusing method. Then, albumin ratio (CSF albumin/serum albumin concentration × 10^-3^), and IgG ratio (CSF IgG/serum IgG concentration × 10^-3^), as additional measures of BCSFB and BBB permeability, were calculated [7]. PD patients were then divided into two groups according to H&Y staging, considering 2 as the grouping cut-off score to compare early with advanced PD subjects [10]. All procedures were carried out with the appropriate understanding and written consent of the subjects. The research protocol had been previously approved by the Ethical Committee of Policlinico Tor Vergata Foundation.

**Table 1:** Clinical data and cerebrospinal fluid (CSF) findings in Parkinson’s disease (PD) patients and controls

	**CTRL**	**IND**	**PD 1-2**	**PD 2.5-4**
No. of patients	47	11	46	27
Age in years	60.4 ± 10.9 (45.0 to 83.0)	59.2 ± 9.0 (45.0 to 75.0)	60.3 ± 9.2 (44.0 to 77.0)	63.9 ± 10.1 (49.0 to 83.0)
Disease duration, months	-	-	25.8 ± 15.1	54.0 ± 41.7
UPDRS III score	-	-	19.0 ± 5.6	38.0 ± 10.6
H&Y stage	-	-	1.6 ± 0.4	3.0 ± 0.5
PD medications	-	-	None: 65.2%	None: 7.4%
			DA: 19.6%	DA: 22.2%
			LD: 4.3%	LD: 33.3%
			LD + DA: 10.9%	LD + DA: 37.1%
Motor fluctuations	-	-	*7*/46	17/27
Albumin ratio (× 10^-3^)	5.2 (2.3 to 13.1)	5.8 (3.4 to 8.1)	5.6 (2.6 to 16.2)	8.2 (3.0 to 21.2)
IgG ratio (× 10^-3^)	2.7 (1.1 to 6.9)	2.8 (1.1 to 4.0)	2.7 (1.1 to 9.5)	3.9 (1.6 to 10.4)

The top half of the table shows clinical data expressed as mean ± SD (minimum to maximum). The lower half shows biochemical values expressed as median (minimum to maximum).

CTRL = control group of healthy subjects; DA = dopamine agonists; H&Y = Hoehn-Yahr; IND = independent group with no neurological disease; LD = levodopa; PD 1–2 = Parkinson’s disease patients with H&Y stage ranging from 1 to 2; PD 2.5-4 = Parkinson’s disease patients with H&Y stage ranging from 2.5 to 4; UPDRS = Unified Parkinson’s Disease Rating Scale.

### Statistical analysis

Data are reported as mean, standard deviation and medians (Table1). Biochemical data, given their non-normal distribution, were separately assessed by one-factor non-parametric Kruskal-Wallis analysis of variance (ANOVA) for four groups. In case of significance, Mann–Whitney test was used to perform multiple comparisons; a *post hoc* Bonferroni correction was applied considering *P* <0.016 as statistically significant. The *χ*^2^ test or Fisher exact test (if there were less than five observations) were used for frequency data, in order to rule out the possibility that differences among groups could depend on random causality. *P* values less than 0.05 were considered significant. Analysis was performed using SigmaStat 3.5 software (Systat Software, Inc., Point Richmond, CA, USA).

## Results

Among 102 PD patients screened, 29 did not meet eligible criteria. Specifically, 11 subjects were excluded because of low MMSE scores, 8 reported intense low-back pain and 10 had confluent cerebral infarctions on MRI. Thus, we analyzed CSF/serum data from 73 PD patients, in comparison with control subjects. We found a significant difference in CSF/serum albumin ratio (*P* = 0.02) between the two groups. According to H&Y staging [10], PD patients (Table1) were then divided into 2 groups: 46 patients with staging between 1 and 2 (PD 1–2) and 27 subjects ranging between 2.5 to 4 H&Y staging (PD 2.5-4). PD 1–2 group showed a mean disease duration of 25.8 ± 15.1 months and a mean UPDRS III score of 19.0 ± 5.6, whereas PD 2.5-4 group had a mean disease duration of 54.0 ± 41.7 months and a mean UPDRS III score of 38.0 ± 10.6. The mean ages of PD 1–2 and 2.5-4 patients (Table1) did not significantly differ (*P* = 0.13).

**Figure 1:**
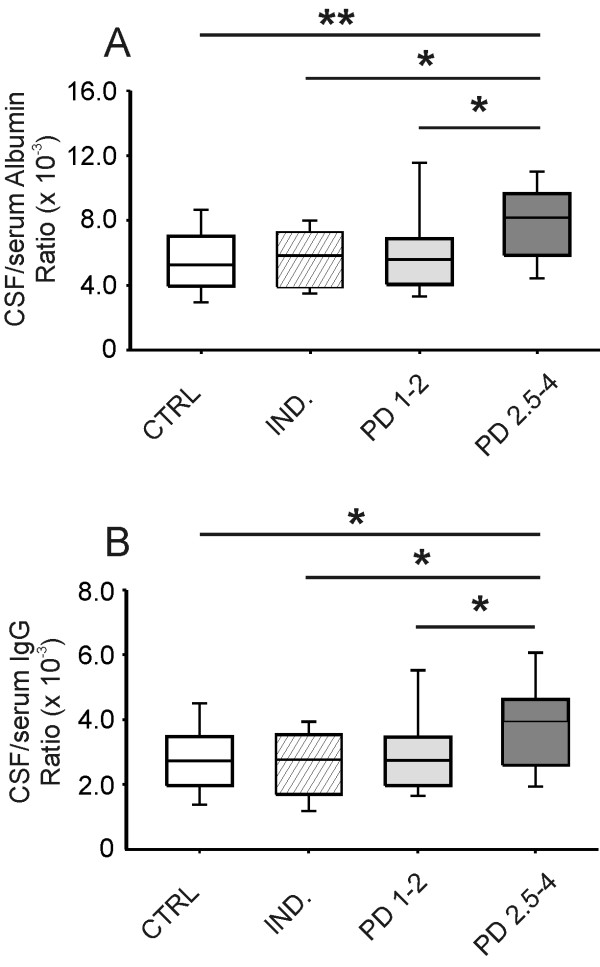
**Albumin and IgG ratios in Parkinson’s disease (PD) and control groups.** Boxplots showing medians and percentiles referred to albumin **(A)** and IgG **(B)** ratios in PD subgroups (PD 1–2, PD 2.5-4) and control subjects (CTRL, IND). Statistically significant differences between groups are reported (*P <0.01; **P <0.001).

Albumin (*P* = 0.002) and IgG (*P* = 0.008) ratios were significantly different in the examined groups. PD 2.5-4 patients differed from CTRL, IND and PD 1–2 groups both in albumin (*P* <0.001, *P* <0.01 and *P* = 0.002, respectively) and IgG ratio values (*P* = 0.002, *P* <0.01 and *P* = 0.005, respectively). Conversely, no statistically significant difference (*P* >0.05) between PD 1–2 and CTRL, IND groups was found (Figure1).

Then, we identified the normal cut-off value for AR (8.3 × 10^-3^), which corresponds to the 90th percentile of AR values in the CTRL group. The number of PD 2.5-4 patients with AR >8.3 × 10^-3^ was higher than that of PD 1–2 patients (*P* = 0.015), thus confirming that the difference between PD groups is not due to random causality. A possible effect of dopaminergic therapy on AR values was assessed by comparing patients with and without pharmacological treatment. The number of treated patients with AR >8.3 × 10^-3^ was higher than that of untreated patients (*P* = 0.035).

## Discussion

The robust effort in search for biomarkers that might help monitoring degenerative processes has led to a renewed interest and application of lumbar puncture for research purposes in a variety of neurodegenerative diseases. CSF concentrations of protein produced within the CNS, such tau protein or α-synuclein, which is also abundantly expressed in red blood cells [11], do not depend on CNS barrier permeability [8]. However, measurement of promising markers of neurodegeneration could be affected by integrity of BBB and possible dysfunctions of BCSFB. This issue is of relevance especially considering that changes in BBB permeability may occur in advanced PD [1,3], and changes have also been demonstrated in PD animal models [4,5]. Although the main cause of PD remains still unclear, several reports suggest that microglial activation, reactive astrocytes, peripheral immune cells infiltration may be implicated in the development of the disease [2,12,13]. Moreover, an increase of brain barrier permeability could allow other elements such as complement, toxins, and metals, normally excluded from the central compartment, to bypass BBB and potentially contribute to the progression of disease. Notably, a BBB dysfunction would likely contribute to alter also ion balance, disrupt transport system, for example, P-glycoprotein [3,14] or Na^+^-dependent levodopa pump activity [15], and potentially impair enzymatic constituents of the barrier.

In our study, we found AR values to be within the normal range in early-staging PD patients, in accordance with previous data [16], whereas they were abnormally high in more advanced phases of the disease. AR is currently recognized as the most reliable marker of BCSFB permeability [7,8], but the BBB also contributes partially to CSF volume and composition [6]. Therefore, an increase of high molecular proteins in the CSF not only indicates BCSFB dysfunction but may also represent an index for BBB impairment. A hypothetical inflammation-dependent and locoregional increase in BBB permeability or a decrease of CSF flow rate by a failure of choroidal Na-K ATPase [17] might explain the increase of blood-derived proteins in the CSF. BBB dysfunction, which is likely sustained by neuroinflammation [2-5], BCSFB hypofunction and decreased CSF flow rate [18], could be meaningful phenomena occurring in advanced stages of PD. However, it is questionable whether such abnormalities play a role as trigger events, participate in the pathogenic process, or are simply consequences of degeneration. Of note, in neurodegenerative diseases, a barrier compromise might also have significant implications for drug delivery, even if BBB alterations do not develop throughout an affected structure in which neurodegeneration occurs, but rather in small localized areas within the structure [5]. Motor complications, such as dyskinesias, that become manifest in advanced stages of the disease after chronic levodopa treatment, could be related to inhomogeneous drug delivery as a result of barrier compromise, or to a reduced capacity of the Na^+^ dependent transport system to remove levodopa from the brain extracellular fluid. The latter hypothesis well fits with a previous study on high levodopa availability in advanced PD patients [19].

## Conclusions

In summary, future neuroimaging studies, along with analyses of proteins that are differently concentrated in the CSF on the basis of the CSF flow rate [8], will clarify the open issues generated by the present study. More insights into the function of CNS barriers could allow the identification of subsets of patients with different responses to drugs, leading to better-tailored therapies.

## Competing interests

The authors declare that they have no competing interests.

## Authors’ contributions

VP, DF and AP designed the study and wrote the paper. VP and MP enrolled the patients and performed clinical assessment. SN collected CSF samples for biochemical analysis and revised the manuscript. VP and SN carried out offline CSF data analysis and performed biostatistical comparisons. AS and DF gave their advice about the recruitment of patients and interpretation of data. PS supervised the whole investigation and revised the manuscript. All authors read and approved the final manuscript.
